# ﻿*Sileneophioglossa* (Caryophyllaceae, Sileneae), a new species from southwest China

**DOI:** 10.3897/phytokeys.225.98247

**Published:** 2023-04-26

**Authors:** Feng Yang, Ting-Ting Wang, Yue-Hua Wang, Huan-Chong Wang

**Affiliations:** 1 School of Life Sciences, Yunnan University, Kunming 650091, China Yunnan University Kunming China; 2 School of Ecology and Environmental Science, Yunnan University, Kunming 650091, China Yunnan University Kunming China; 3 Herbarium of Yunnan University, Kunming 650091, Yunnan, China Yunnan University Kunming China

**Keywords:** Conservation assessment, endemism, ITS sequence, *Silenephoenicodonta*, *Silene* sect. *Cucubaloides*

## Abstract

*Sileneophioglossa* Huan C. Wang & Feng Yang, a new species of Caryophyllaceae, is here described and illustrated based on morphological and molecular evidence. The new species was found in Sichuan and Yunnan provinces, southwest China. Phylogenetic analysis based on ITS sequences showed this new species belongs to section Cucubaloides. Morphologically, it resembles *S.phoenicodonta* and *S.viscidula*, which were also found in the southwest China, but clearly differs from the latter two species by having 5–7 mm long calyces with sparsely hirtellous and short glandular hairs, white petals, linear limbs and lobes, and absent or oblong-linear coronal scales. A distribution map and a table with morphological diagnostic characters of new species and its closest relatives are provided, as well as a preliminary conservation assessment of *S.ophioglossa* under the IUCN criteria.

## ﻿Introduction

The genus *Silene* L. (Sileneae DC., Caryophyllaceae Juss.), with 700 to 870 species (Mabberley 2017; Jafari et al. 2020) is mainly distributed in temperate regions of the Northern Hemisphere (Greuter 1995; Oxelman and Lidén 1995; Zhou et al. 2001). The center of its diversity is considered to be in western Asia and the Mediterranean area, but areas of central Asia are also highly diverse (Jafari et al. 2020). There has been considerable controversy regarding delimitation of the genus *Silene*. Recent molecular studies have clearly demonstrated that *Silene* (in the traditional sense), is paraphyletic since *Lychnis* L., *Atocion* Adans. and *Viscaria* Bernh. are nested within it (Jafari et al. 2020). Some authors suggested lumping most members of Sileneae into the genus (e.g. Greuter 1995; Desfeux and Lejeune 1996; Jafari et al. 2020). Conversely, other authors (e.g. Oxelman and Lidén 1995; Oxelman et al. 1997, 2001; Popp and Oxelman 2004; Frajman et al. 2009a, b; Greenberg and Donoghue 2011) preferred to separate *Agrostemma* L., *Eudianthe* (Rchb.) Rchb., *Heliosperma* Rchb., *Petrocoptis* A. Braun, *Atocion* and *Viscaria*.

The first comprehensive revision of the genus *Silene* in China was made by Tang (1996) who recognized 112 species, 2 subspecies and 17 varieties. In the most recent treatment by Zhou et al. (2001), 110 species were accepted, 67 of which are endemic to China (endemism ratio of *Silene* in China is about 61%). More recently, three additional species of *Silene* were described or recorded from southwest China by Lin et al. (2019) and Yang et al. (2022 a, b); these findings highlight the need for continued field exploration and taxonomical research in the region.

During our field surveys and the herbarium studies for a taxonomic revision of *Silene* in the Sino-Himalayan region, an interesting plant was repeatedly encountered, but one that does not fit with any previously described species. Comparison with related species demonstrates that this plant actually represents a distinct species hitherto not described. Therefore, it is described as a new species herein and named as *Sileneophioglossa* Huan C. Wang & Feng Yang.

## ﻿Material and methods

### ﻿Morphological analyses

The study followed the normal practice of plant taxonomic survey and herbarium taxonomy. Morphological studies of the new species were based on observation of living plants and specimens from Yunnan and Sichuan, southwest China. Digital images available at the JSTOR Global Plants (http://plants.jstor.org/) and at the Chinese Virtual Herbarium (http://www.cvh.ac.cn/), as well the collections housed at CDBI, KUN, PE, PYU, XTBG and YUKU were examined and compared with the new species. Pertinent taxonomic literature (e.g. Wu 1993; Zhuang 1995; Tang 1996; Zhou et al. 2001) was consulted. Morphological studies were carried out on dried material under a stereomicroscope (Olympus SZX2, Tokyo, Japan) and measurements were made using a ruler and a metric vernier caliper.

### ﻿Seed micromorphology

Mature seed samples were directly adhered to carbon adhesive tape. Then they were coated with gold palladium using a BAL-TEC SCD 005 cool sputter coater (BAL-TEC AG., Liechtenstein) at Yunnan University, Kunming, China. Observations were conducted using a QUANTA 200 scanning electron microscope (SEM) (FEI Co., U. S. A.) at 20.0 KV.

### ﻿Phylogenetic study

To determine the phylogenetic position of the putative new species, the internal transcribed spacer region (ITS) of the nuclear ribosomal DNA was used as the molecular marker. Total genomic DNA of this new species and *S.phoenicodonta* were extracted from silica-gel dried leaves using the DNA secure plant kit (TIANGEN, Beijing, China). The PCR protocols followed Lin et al. (2019). The ITS primers used were ITS1 and ITS4, as described by White et al. (1990) and Urbatsch et al. (2000). The PCR products were bidirectionally sequenced with the same primers used for PCR amplifications in an ABI 3730xL DNA Analyzer (Applied Biosystems) at Kunming Branch of Beijing Qingke Biotechnology Co., Ltd. (Yunnan, China).

We used a total of 70 taxa of *Silene* as ingroups, being representatives of most sections of *Silene*, *Heliospermaoliverae* Niketić et Stevan., *Atocionarmeria* (Fedor.) Fedor. and *Petrocoptispyrenaica* A. Braun as outgroups following the previous phylogenetic analyses (Jafari et al. 2020). A total of 73-taxon data sets, including six newly published sequences, were obtained. Voucher specimen and GenBank accession information for taxa are listed in Appendix 1.

All sequences were aligned with MAFFT (Katoh and Standley 2013) using ‘auto’ strategy and normal alignment mode. Gap sites were removed with trimAl (Capella‐Gutiérrez et al. 2009) using “-automated1” command. The best-fitting substitution models SYM+I+G model for Bayesian inference were selected using ModelFinder (Kalyaanamoorthy et al. 2017) in BIC criterion. MrBayes 3.2.6 (Ronquist et al. 2012) was used to conduct Bayesian phylogenetic analyses. Runs were performed for 5 million generations with sampling of trees every 500 generations. The initial 25% of sampled data were discarded as burn-in.

## ﻿Results and taxonomic treatment

### ﻿ Silene ophioglossa

Taxon classificationPlantaeCaryophyllalesCaryophyllaceae

Huan C. Wang & Feng Yang sp. nov.

51F0024F-3E33-57C9-96C0-9A4D025BA253

urn:lsid:ipni.org:names:77318297-1

Figs 1 , 2

#### Type.

China. Yunnan Province: Binchuan County, Pingchuan Town, Maojiaoshan Mountain, alt. 2198 m, 25°58'13.6"N, 100°42'8.28"E, under a walnut forest by a ravine stream, 17 June 2022, *F. Yang et al. BC17342* (holotype YUKU-02074705!; isotypes YUKU-02074706!, PE!, HITBC!).

**Figure 1.**
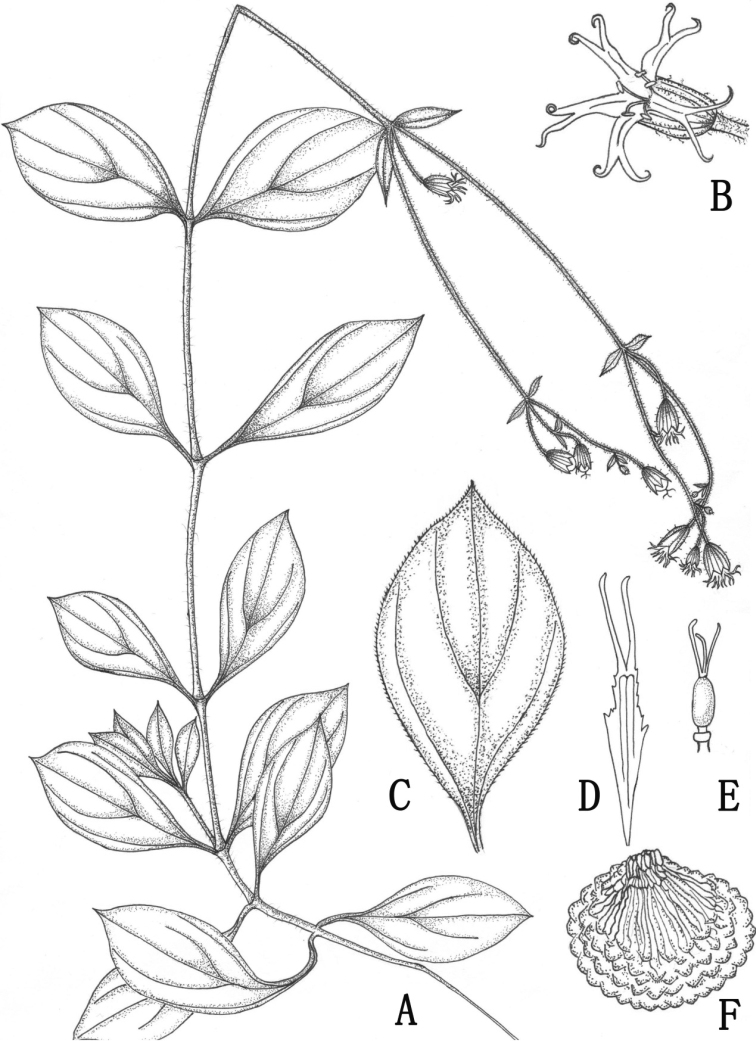
*Sileneophioglossa* sp. nov. (Drawn by Tingting Wang) **A** habit **B** flower (front view) **C** adaxial surface of leaf **D** petal **E** pistil and androgynophore **F** seed.

**Figure 2.**
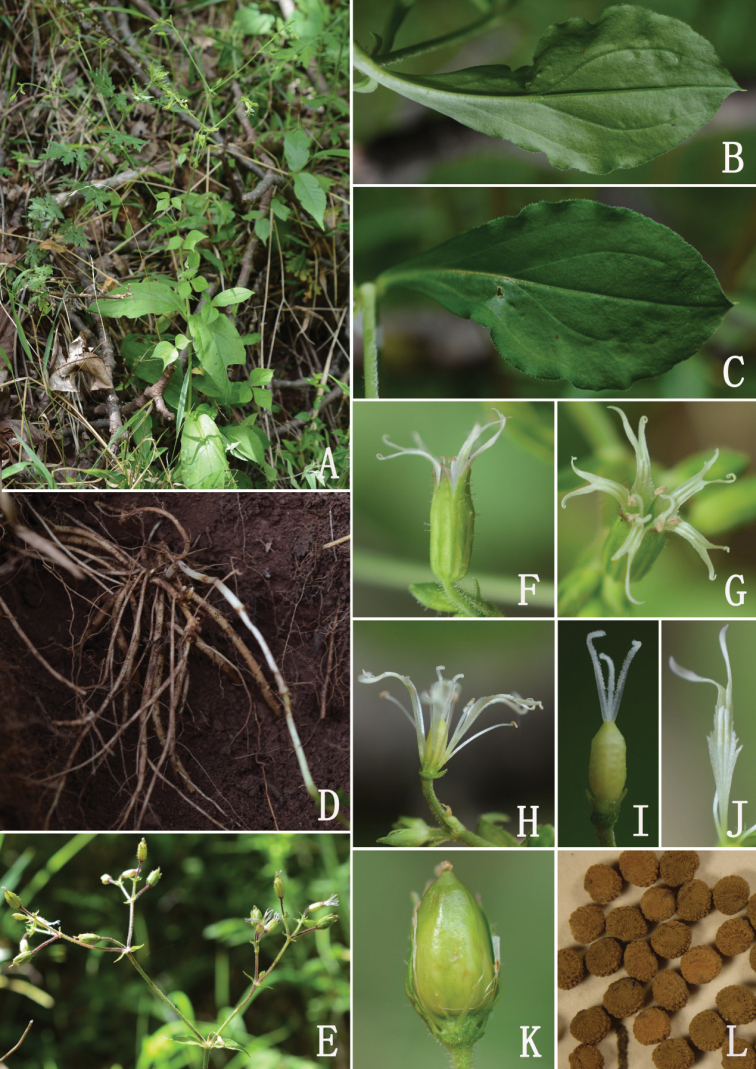
*Sileneophioglossa* sp. nov. (Photographed by F. Yang) **A** habit **B** abaxial surface of leaf **C** adaxial surface of leaf **D** roots **E** dichasial cymes **F** flower (side view, showing the calyx) **G** flower (front view) **H** dissected flower (showing the androgynophore and claws) **I** pistil and androgynophore **J** petal (showing the claw, auricles and coronal scales) **K** immature capsule **L** seeds.

#### Diagnosis.

**Figure 3.**
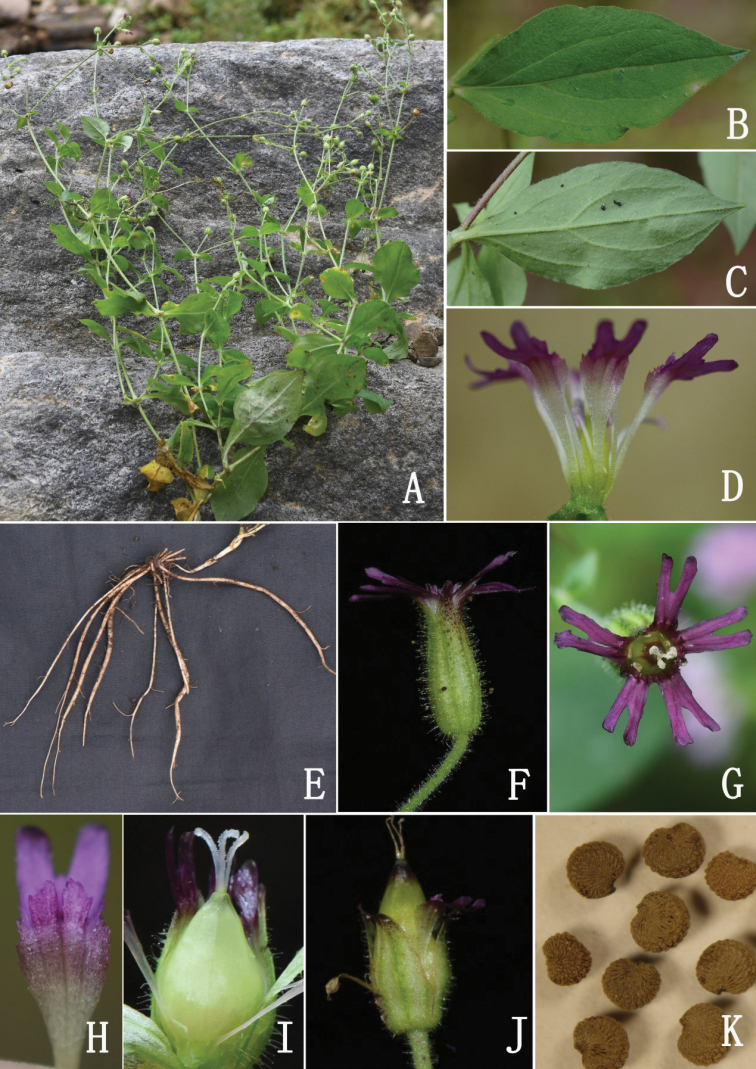
*Silenephoenicodonta* (Photographed by F. Yang and H. C. Wang) **A** habit **B** adaxial surface of leaf **C** abaxial surface of leaf **D** dissected flower (showing the androgynophore and claws) **E** roots **F** flower (side view, showing the calyx and pedicel) **G** flower (front view) **H** petal (showing the claw, auricles and coronal scales) **I** styles and immature capsule **J** calyx after anthesis **K** seeds.

*Sileneophioglossa* is morphologically similar to *S.phoenicodonta* (Fig. 3), but clearly differs from the latter in having 5–7 mm (vs. 6–8 mm) long calyces sparsely hirtellous and short glandular hairs (vs. densely hirtellous and with short glandular hairs), white (vs. dark violet) petals, linear (vs. obovate) limbs and linear (vs. ovate or nearly band-shaped) lobes, absent or oblong-linear (vs. orbicular-linear) coronal scales.

#### Etymology.

The specific epithet “*ophioglossa*” is derived from the Greek words “*ophis*” (meaning snake) and “*glossa*” (meaning tongue), which refer to the petal lobes of this new species which resemble the tongue of a snake.

#### Description.

Herbs perennial. Roots numerous, clustered, cylindric, fleshy. Stems sparsely caespitose, ascending to sprawling, 30–80 cm long, slender, multibranched, with sparsely pubescent. Leaves ovate-elliptic or obovate-elliptic, 3–9 (–15) cm long, 1–4 cm wide, base cuneate or attenuate into petiole, apex acute, both surfaces puberulent to subglabrous, margin entire, minutely ciliate, lateral veins 2 pairs, midrib and lateral veins prominent abaxially. Dichasial cymes terminal, diffuse; peduncle 1–15 cm long, densely glandular-pilose. Pedicels densely glandular-pilose and sparsely eglandular villous, subequaling or longer than calyx; bracts ovate-lanceolate, apex acuminate. Calyx tubular-campanulate, 5–7 mm long, 2–3 mm in diameter, longitudinal veins green or violet, cohering at apex, sparsely hirtellous and with short glandular hairs, inflated after anthesis, 6–7 mm long, 4–5 mm in diameter in fruit stage; calyx teeth ovate-triangular, green or violet, ca. 1 mm long, apex acute, margin ciliate. Androgynophore ca. 1 mm long, glabrous. Petals white, 1.0–1.2 cm long; claws equaling to calyx, oblanceolate, glabrous, inflated above, margin erose; limbs linear, deeply bifid to middle, lobes linear, apex usually curly, without side lobe; coronal scales absent or oblong-linear, small, entire or emarginated at apex. Stamens 10, included in calyx tube; filaments glabrous, 5–7 mm long. Styles 3, included or slightly exserted beyond calyx. Capsule broadly ovoid, 7–9 mm long, 4–5 mm in diameter, slightly longer than calyx. Seeds dark brown, globose-reniform, ca. 1 mm long, tuberculate.

#### Molecular phylogenetics.

The ITS sequence region of *Sileneophioglossa* comprises 687 and 821 base pairs with a GC content of 53.3% and 54.4%. The alignment of 73 ITS sequences resulted in a matrix of 582 total characters, 350 of which are constant, 58 of the variable characters are singleton sites and 174 characters are parsimony-informative sites.

**Figure 4.**
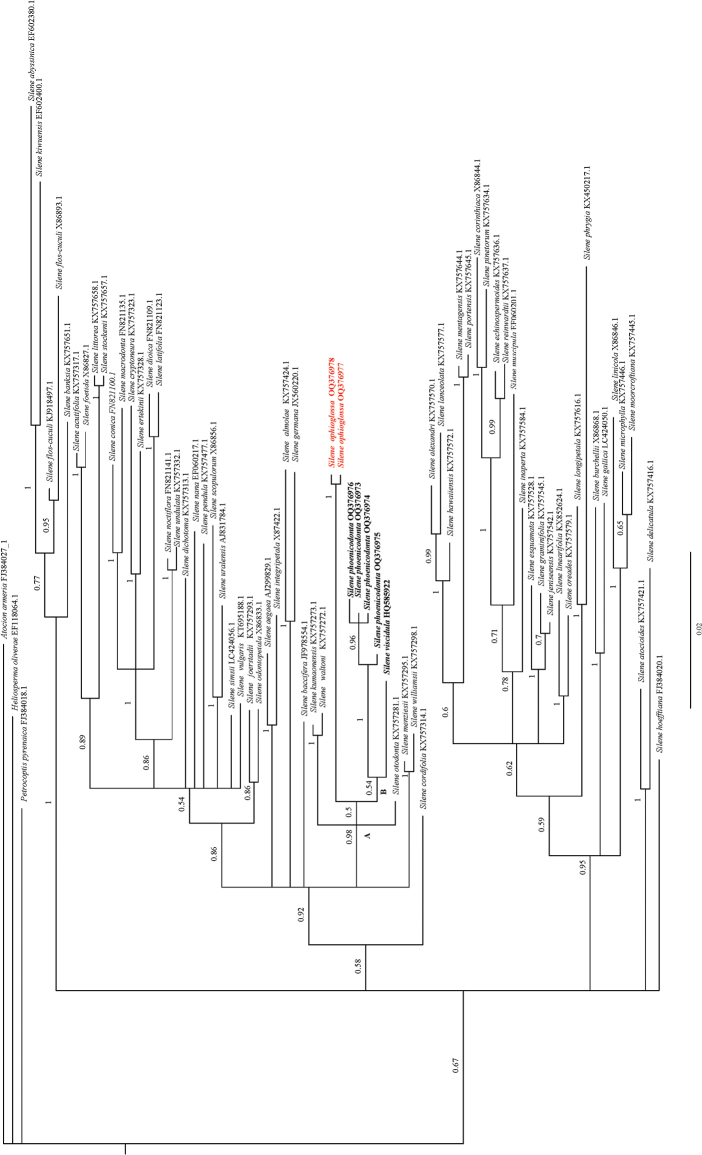
Bayesian inference tree of *Silene* based on ITS sequences showing phylogenetic placements of *S.ophioglossa*. Bayesian posterior probabilities are shown near the nodes. *Sileneophioglossa*, *S.viscidula* and *S.phoenicodonta* are marked in bold type, and *S.ophioglossa* is highlighted by using red colored text.

Phylogenetic analyses using ITS sequences uncovered that the new species, *S.ophioglossa*, belongs to a clade A representing S.sect.Cucubaloides Edgeworth & Hook. f. which was recircumscribed by Jafari et al. (2020) in their recent studies (Fig. 4). This placement is also supported by its morphological characters, such as the ascending to sprawling, multibranched stems, ovate-elliptic or obovate-elliptic leaves, lax dichasial cymes and tubular-campanulate calyces. In the phylogenetic tree (Fig. 4), two sequences from *S.ophioglossa* constituted a monophyletic lineage with maximum support, and it is sister to a small subclade B that includes *S.phoenicodonta* and *S.viscidula*. This close relationship is also supported by their morphological similarity.

#### Seed micromorphology.

**Figure 5.**
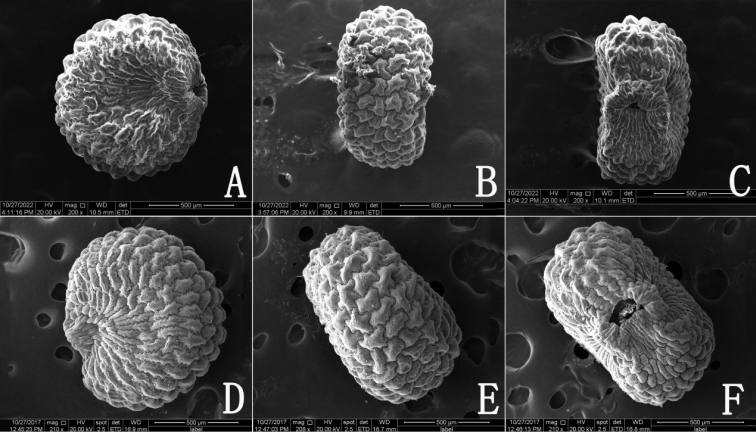
SEM micrographs of seed morphology in *Sileneophioglossa* (voucher specimen: Q. P. Wang et al. XY7908, **A–C**) and *S.phoenicodonta* (voucher specimen: D. Qiao DC2575, **D–F**). **A, D** lateral surface **B, E** dorsal surface **C, F** ventral surface.

Seeds of *Sileneophioglossa* are dark brown when mature, globose-reniform in shape, 0.94–1.12 mm long, 0.79–0.94 mm wide. The lateral surface of seed is concave. The dorsal surface is flat, ca. 0.57 mm wide. Its seed coat is formed by elongate epidermis cells with S-undulate and V-undulate anticlinal walls. The periclinal walls are convex and have granulate-papillate ornamentation (Fig. 5A–C).

#### Phenology.

Flowering and fruiting from June to September.

#### Distribution and habitat.

**Figure 6.**
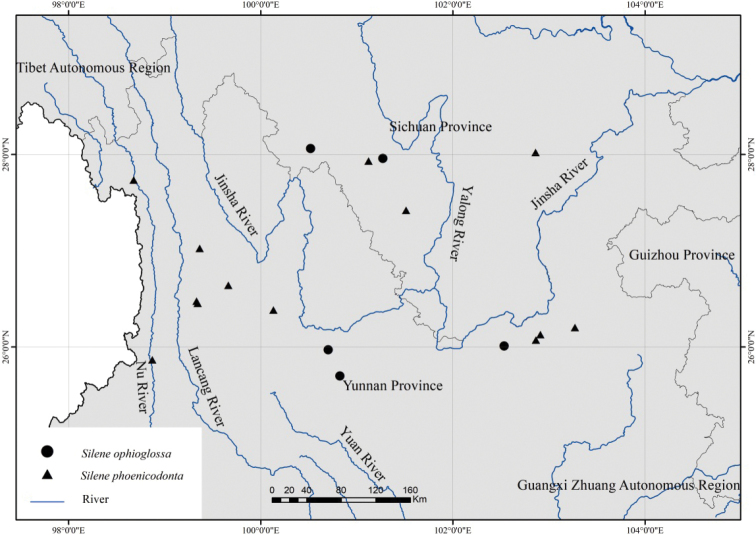
Distribution of *Sileneophioglossa* and *S.phoenicodonta* in southwest China.

*Sileneophioglossa* is endemic to southwest China, where it has been collected from western Sichuan and north Yunnan (Fig. 6). Currently, it seems to be restricted to the Jinsha River basin. *Sileneophioglossa* usually occurs at elevations ranging from 2000–3000 meters and grows in thickets or at forest margins, and its association includes *Lepisoruspalmatopedatus* (Baker) C. F. Zhao, R. Wei et X. C. Zhang, *Lysimachiachristiniae* Hance, *Corydalistriternatifolia* C. Y. Wu, *Indigoferapendula* Franch., LysimachiastenosepalaHemsl.var.flavescens Chen et C. M. Hu, *Circaeacordata* Royle, *Quercusacutissima* Carr. and *Campylotropisteretiracemosa* P. C. Li et C. J. Chen.

#### Conservation status.

*Sileneophioglossa* is known from five localities and has been found in thickets or at forest margins. However, we actually only investigated two points, and didn’t have enough information about its distribution, abundance, or threats to this species. More information is needed for assignment of its conservation status. Therefore, we choose to assign this new species to the category data deficient (DD) following the IUCN guidelines (IUCN 2012, 2022).

#### Taxonomic notes.

Morphologically and seed micromorphologically, *Sileneophioglossa* is most similar to *S.phoenicodonta* (Fig. 3), a species also distributed in southwest China (Fig. 6). They share ascending to sprawling, multibranched stems, ovate-elliptic or obovate-elliptic leaves, dichasial cymes, 3 styles and globose-reniform seeds (Fig. 5), but the new species is distinguishable from the latter by its calyces 5–7 mm (vs. 6–8 mm) long, sparsely (vs. densely) hirtellous and with short glandular hairs, petals white (vs. dark violet), limbs linear (vs. obovate), lobes linear (vs. ovate or nearly band-shaped) and coronal scales absent or oblong-linear (vs. orbicular-linear).

*Sileneophioglossa* is somewhat close to *S.viscidula*, an endemic species found from southwestern China. Nevertheless, *S.ophioglossa* differs from *S.viscidula* in having ovate-elliptic or obovate-elliptic (vs. elliptic or elliptic-oblanceolate) leaves, diffuse (vs. compact, 3–7 (–15)-flowered) dichasia, 5–7 mm long (vs. 7–10 mm long) calyces, sparsely hirtellous and with short glandular hairs (vs. densely glandular hairs), white (vs. pale pink or white) petals, linear (vs. broadly obovate) limbs, linear (vs. ovate or square) lobes, absent or oblong-linear (vs. flabellate) coronal scales. A detailed morphological comparison between these three species is summarized in Table 1.

**Table 1.** Morphological comparison of *Sileneophioglossa*, *S.phoenicodonta* and *S.viscidula*.

Characters	Species		
	*S.ophioglossa*	*S.phoenicodonta*	*S.viscidula*
**Roots**	clustered, cylindric	clustered, cylindric	clustered, fusiform
**Leaves**	ovate-elliptic or obovate-elliptic	ovate-elliptic or obovate-elliptic	elliptic or elliptic-oblanceolate
**Inflorescence**	dichasial cymes, diffuse	dichasia diffuse, few flowered	dichasial cymes, 3–7 (–15)-flowered, compact
**Calyces**	5–7 mm long, outside sparsely hirtellous and with short glandular hairs	6–8 mm long, outside densely hirtellous and with short glandular hairs	7–10 mm long, outside with dense glandular hairs
**Petals**	white	dark violet	pale pink or white
**Limbs**	linear, deeply bifid to middle	obovate, deeply bifid to middle	broadly obovate, shallowly 2-lobed
**Lobes of petals**	linear, apex usually curly, without side lobe	ovate or nearly band-shaped, sometimes with one inconspicuous tooth on each lateral side	ovate or square, entire, sometimes with one inconspicuous tooth on each lateral side
**Coronal scales**	absent or oblong-linear, entire or emarginated at apex	Present, orbicular-linear, laciniate at apex	Present, flabellate, laciniate at apex

#### Additional specimens examined (Paratypes).

**China. Sichaun**: Muli County, Qiaowa Town, Chutouwan village, under the shady and moist thickets by the river, alt. 2600 m, 28 June 1978, *Y. B. Yang 7097* (CDBI-0020627, CDBI-0020628); Muli County, Ninglang village, under *Tsuga* forest on the mountain slope, alt. 3000 m, 25 September 1983, *Qinghai-Tibet Expedition 14350* (KUN-0514408, KUN-0514409). **Yunnan**: Luquan County, Sayingpan Town, Sayongshan Mountain, at evergreen broad-leaved forest margins, alt. 2400 m, 21 June 1965, *W. M. Zhu & Y. M. Feng 00584* (YUKU-02006723); Xiangyun County, Midian Town, Yemaoshan Mountain, alt. 2300 m, 25°41'52.12"N, 100°49'26.69"E, 24 July 2018, *Xiangyun Medicinal Plant Investigation Team 5329230617* (YUKU-02074711, YUKU-02074712, YUKU-02074713) and *F. Yang et al. XY8075* (YUKU-02074710); same location, 26 August 2019, *Q. P. Wang et al. XY7908* (YUKU-02074714, YUKU-02074715).

*Silenephoenicodonta*. **China. Sichaun**: Zhaojue County, Sikai Town, thickets, alt. 2400 m, 30 June 1976, *Sichuan Vegetation Team 12763* (PE-00580695, CDBI-0020501). **Yunnan**: Huizhe County, Dahai village, Dahaicaoshan, ca. 2 km from Xiaoxiniu, Dabaping, alpine meadows, 103°16'10.50"E, 26°12'6.19"N, alt. 3433 m, 24 July 2018, *H. Tang TH2018046* (KUN-1481546); Dongchuan District, Xueling Scenic Area, 29 August 2017, *D. Qiao DC2575* (YUKU-02074716); Heqing County, Songgui Town, Maershan Mountain, on the path from Chamujing to Zhulinkou, under forest, roadsides, 100°7'53.68"E, 26°23'2.58"N, alt. 2578 m, 5 August 2018, *H. Tang TH2018087* (KUN-1481543); Jianchuan County, Shizhongshan Mountain, July 1987, *S. Y. Bao 401* (KUN-0531671); Zhengkang County, Snow Range, in grassy slope, alt. 2600 m, 22 July 1938, *T. T. Yu 16881* (PE-00558309, KUN-0514405).

*Sileneviscidula*. **China. Sichaun**: Yanbian County, Dapingzi District, Baoshishan, limestone mountainous region, at 2700 m, 29 June 1983, *Qinghai-Tibet Team 11677* (KUN-0514407). **Guizhou**: Weining County, Mazha Town, Gali village, Mabaidashan, 12 July 1959, *Bijie Team 191* (PE-00581476). **Yunnan**: Luquan County, Kedu Town, Dianwei village, grassy slope, at 2500 m, 25 October 1940, *Y. B Chang 347* (IBSC-0149532); Dongchuan District, November 1906, *E. E. Maire 87* (E-00109656); Mengzi, Yangliuhe village, sparse forest, at 1720 m, 30 July 1958, *Y. Y. Hu & S. K. Wen 580546* (KUN-0514415).

## Supplementary Material

XML Treatment for Silene ophioglossa

## ﻿Appendix 1

**Table A1.** Voucher information and GenBank accession of species used in our study.

GenBank	Species	Voucher information	Herbarium
AJ299829	*Sileneaegaea* Oxelman	Christodoulakis 2826	UPA
AJ831784	*Sileneuralensis* (Rupr.) Bocquet	Inger Skrede SUP02-38-08	UPS
EF060201	*Silenemuscipula* L.	Bengt Oxelman 1780	GB
EF060217	*Silenenana* Kar. Et Kir.	Kereverzova and Mekeda 1976.V.5	LECB
EF118064	*Heliospermaoliverae* Niketić et Stevan.	LJU 137738	
EF602380	*Sileneabyssinica* (Hochst.) H.Neumayer	P. Tuley 2091	K
EF602400	*Silenekiwuensis* (Fr.) F. Jafari, Oxelman et Rabeler	A. Gizaw AFR561	ETH
FJ384018	*Petrocoptispyrenaica* A. Braun	Schneeweiss et al. 6549	WU
FJ384020	*Silenehoefftiana* Fisch. ex C. A. Mey.	Menitskiy and Portenier 1987.VIII4	LE
FJ384027	*Atocionarmeria* (Fedor.) Fedor.	B. Frajman and M. Turjak 136972	LJU
FN821100	*Sileneconica* L.	Erixon 70	UPS
FN821109	*Silenedioica* (L.) Clairv.	H. C. Prentice D130-2	GB
FN821123	*Silenelatifolia* Poir.	H. C. Prentice L80-12	GB
FN821135	*Silenemacrodonta* Boiss.	Bengt Oxelman 2441	GB
FN821141	*Silenenoctiflora* L.	H. C. Prentice N2-2	
JF978554	*Silenebaccifera* (L.) Roth	Z387	
JX560220	*Silenegermana* J. Gay ex Coss.		
KJ918497	*Sileneflos-cuculi* (L.) Clairv	S-99	
KT695188	*Silenevulgaris* (Moench) Garcke	BIOUG24049-D08	
KX450217	*Silenephrygia* Boiss.	MUFE12337	
KX757272	*Silenewaltoni* F. N. Williams	G. and S. Miehe 03-048-12	Miehe
KX757273	*Silenekumaonensis* F. N. Williams	G. and S. Miehe 01-109-08	Miehe
KX757281	*Sileneotodonta* Franch.	Miehe/Huang/Otsu/Tunsu 97-070-18	Miehe
KX757293	*Silenejoerstadii* Wendelbo	Hedge and Wendelbo W 8992	GB
KX757295	*Silenemenziesii* Hook.	A.R. Kruckeberg 2830	WTU
KX757298	*Silenewilliamsii* Britton	C. Brochmann and H. H Grundt	
KX757313	*Silenedichotoma* Ehrh.	Till s.n. 17.7.2004	WU
KX757314	*Silenecordifolia* All.	Lippert and Merxmueller 17265	
KX757317	*Sileneacutifolia* All.	Rothmaler 13691	S
KX757323	*Silenecryptoneura* Stapf	Bengt Oxelman 2524	GB
KX757328	*Sileneertekinii* Aydin et Oxelman	Zeynep Aydin 31	
KX757332	*Sileneundulata* Aiton	Bayliss, Roy Douglas Abbot BS7700	M
KX757416	*Silenedelicatula* Boiss.	Bengt Oxelman 2456	GB
KX757421	*Sileneatocioides* Boiss.	Bengt Oxelman 1627	GB
KX757424	*Silenealmolae* J. Gay ex Coss.	Merxmueller, H. and Lippert, W. 25372	M
KX757445	*Silenemoorcroftiana* Wall. ex Benth.	Ewald and Zetterllund 6278	GB
KX757446	*Silenemicrophylla* Boiss.	Abbas Gholipour 2	
KX757477	*Silenependula* L.	Bengt Oxelman 2291	GB
KX757528	*Sileneesquamata* W. W. Sm.	Podlech, D. 54568	M
KX757542	*Silenejeniseensis* Willd.	H. Solstad and R. Elven 04/1573	O
KX757545	*Silenegraminifolia* Otth	Anja Rautenberg 241	UPS
KX757570	*Silenealexandri* Hillebr.	Wood 6036	US
KX757572	*Silenehawaiiensis* Sherff	MW Chase 2013	K
KX757577	*Silenelanceolata* A.Gray	S. Weller 4	
KX757579	*Sileneoreades* Boiss. et Heldr.	Anja Rautenberg 167	UPS
KX757584	*Sileneinaperta* L.	Silva 1688	LD
KX757616	*Silenelongipetala* Vent.	Grey-Wilson and Hewer 899	GB
KX757634	*Silenepinetorum* Boiss. et Heldr.	Bengt Oxelman et al 2183	GB
KX757636	*Sileneechinospermoides* Hub.-Mor.	Bengt Oxelman 2202	GB
KX757637	*Silenereinwardtii* Roth	T. Engel, Frey and H. Kuerschner 90-223	BSB
KX757644	*Silenementagensis* Coss.	Jahandiez 329	LD
KX757645	*Sileneportensis* L.	Podlech 46825	G
KX757651	*Silenebanksia* (Meerb.) Mabb.	S.J. Enander	S
KX757657	*Silenestockenii* Chater	Holmdahl 1595	GB
KX757658	*Silenelittorea* Brot.	Bengt Oxelman 2589	GB
KX852624	*Silenelinearifolia* Otth	IB-13681	
LC424050	*Silenegallica* L.	27286	TUH
LC424056	*Silenesimsii* F. Jafari, Rabeler et Oxelman	39821	TUH
X86827	*Silenefoetida* Link ex Spreng.	Bengt Oxelman s.n	GB
X86833	*Sileneodontopetala* Fenzl		
X86844	*Silenecorinthiaca* Boiss. et Heldr.	Bengt Oxelman 1951	GB
X86846	*Silenelinicola* C. C. Gmel.	Bengt Oxelman ITS-20605	GB
X86856	*Silenescopulorum* Franch.	KGB 810	GB
X86868	*Sileneburchellii* Otth	Bengt Oxelman 2280	GB
HQ585922	*Sileneviscidula* Franch.	SV6	
X87422	*Sileneintegripetala* Bory et Chaub.	Jagel 48	B
OQ376973	*Silenephoenicodonta* Franch.	H. C. Wang et al. LQ12915	YUKU
OQ376974	*Silenephoenicodonta* Franch.	F. Yang et al. LP4339	YUKU
OQ376975	*Silenephoenicodonta* Franch.	J. L. Liu et al. LQ14001	YUKU
OQ376976	*Silenephoenicodonta* Franch.	H. C. Wang et al. JC4486	YUKU
OQ376977	*Sileneophioglossa* Huan C. Wang et Feng Yang	Xiangyun Med. Pl. Exp. 5329230617	YUKU
OQ376978	*Sileneophioglossa* Huan C. Wang et Feng Yang	Q. P. Wang et al. XY7908	YUKU
